# Supplementary material for: Heterologous Booster Immunization Based on Inactivated SARS-CoV-2 Vaccine Enhances Humoral Immunity and Promotes BCR Repertoire Development
Source: Vaccines (Basel). 2024 Jan 24;12(2):120. doi: 10.3390/vaccines12020120 (PMC10891849; doi:10.3390/vaccines12020120)
Supplement: Supplementary file 1 [file vaccines-12-00120-s001.zip › Supplementary File.pdf]

Figure S1. V-J gene pairing of heavy and light chains.

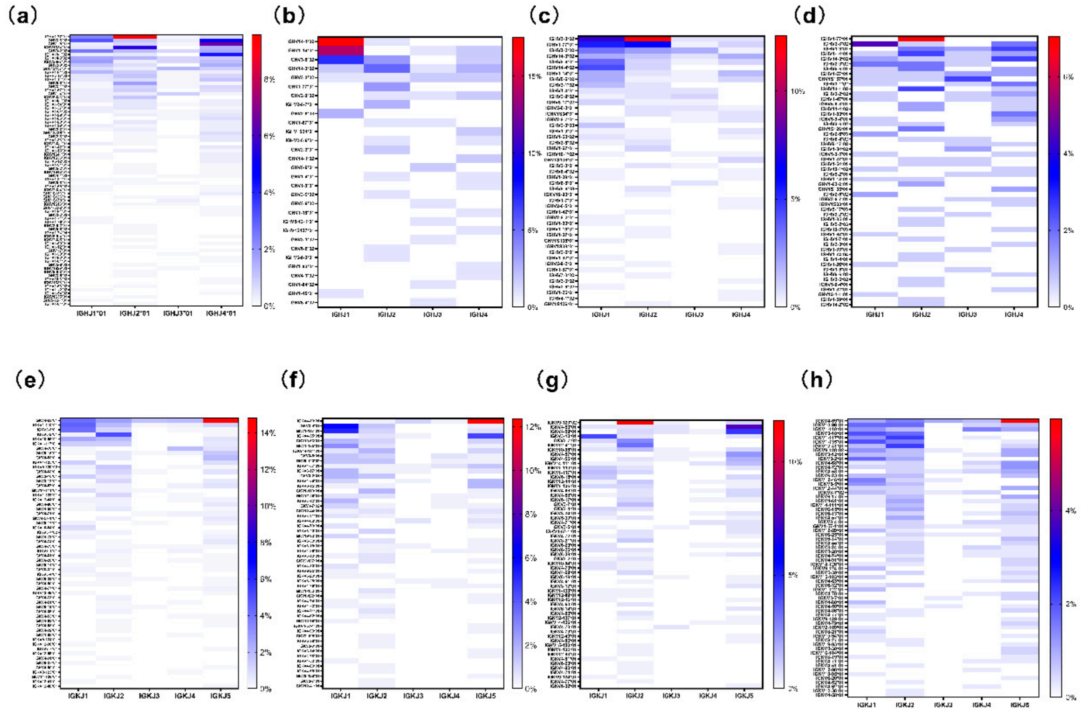

(a-d) V-J gene pairs for heavy chains of groups A-D, respectively. (e-h) V-J gene pairs for light chains of groups A-D, respectively.
